# The chromosome-scale genome of black wolfberry (*Lycium ruthenicum*) provides useful genomic resources for identifying genes related to anthocyanin biosynthesis and disease resistance

**DOI:** 10.1016/j.pld.2025.01.001

**Published:** 2025-01-06

**Authors:** Gulbar Yisilam, Enting Zheng, Chuanning Li, Zhiyong Zhang, Ying Su, Zhenzhou Chu, Pan Li, Xinmin Tian

**Affiliations:** a Key Laboratory of Ecology of Rare and Endangered Species and Environmental Protection (Ministry of Education) & Guangxi Key Laboratory of Landscape Resources Conservation and Sustainable Utilization in Lijiang River Basin, Guangxi Normal University, Guilin 541006, China; b Xinjiang Key Laboratory of Biological Resources and Genetic Engineering, College of Life Science and Technology, Xinjiang University, Urumqi 830046, China; c Laboratory of Systematic & Evolutionary Botany and Biodiversity, College of Life Sciences, Zhejiang University, Hangzhou 310058, China; d Guangxi University Enginering Research Center of Bioinformation and Genetic Improvement of Speciaty Crops, Guangxi Normal University, Guilin 541006, China

**Keywords:** *Lycium ruthenicum*, Genome, Anthocyanin biosynthesis, Gene duplication, Comparative genomics

## Abstract

The black wolfberry (*Lyciumruthenicum*; 2*n* = 2*x* = 24) is an important medicinal plant with ecological and economic value. Its fruits have numerous beneficial pharmacological activities, especially those of anthocyanins, polysaccharides, and alkaloids, and have high nutritional value. However, the lack of available genomic resources for this species has hindered research on its medicinal and evolutionary mechanisms. In this study, we developed the telomere-to-telomere (T2T) nearly gapless genome of *L. ruthenicum* (2.26 Gb) by integrating PacBio HiFi, Nanopore Ultra-Long, and Hi-C technologies. The assembled genome comprised 12 chromosomes with 37,149 protein-coding genes functionally annotated. Approximately 80% of the repetitive sequences were identified, of which long terminal repeats (LTRs) were the most abundant, accounting for 73.01%. The abundance of LTRs might be the main reason for the larger genome of this species compared to that of other *Lycium* species. The species-specific genes of *L. ruthenicum* were related to defense mechanisms, salt tolerance, drought resistance, and oxidative stress, further demonstrating their superior adaptability to arid environments. Based on the assembled genome and fruit transcriptome data, we further constructed an anthocyanin biosynthesis pathway and identified 19 candidate structural genes and seven transcription factors that regulate anthocyanin biosynthesis in the fruit developmental stage of *L. ruthenicum*, most of which were highly expressed at a later stage in fruit development. Furthermore, 154 potential disease resistance-related nucleotide-binding genes have been identified in the *L. ruthenicum* genome. The whole-genome and proximal, dispersed, and tandem duplication genes in the *L. ruthenicum* genome enriched the number of genes involved in anthocyanin synthesis and resistance-related pathways. These results provide an important genetic basis for understanding genome evolution and biosynthesis of pharmacologically active components in the *Lycium* genus.

## Highlights

• This study assembed a nearly gapless telomere-to-telomere (T2T) genome of *Lycium ruthenicum.*
• The abundance of long terminal repeats (LTRs) might be the main reason for the larger genome of this species compared to that of other *Lycium* species.
• The different types duplication genes in the *Lycium ruthenium* genome enriched the number of genes involved in anthocyanin synthesis and resistance-related pathways.

## Graphical abstract

**Figure:**
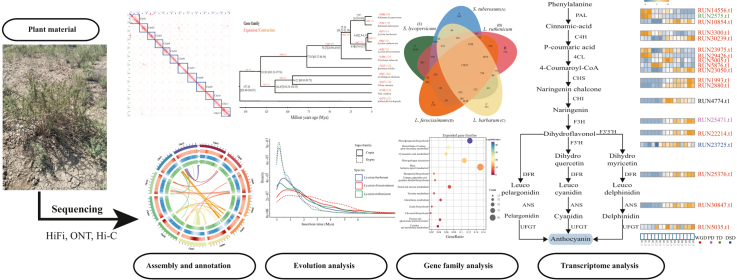

## 1 Introduction

*Lycium ruthenicum* Murr. (Solanaceae), a traditional Chinese medicinal plant also referred to as black wolfberry, is mainly distributed in Central and Western Asia (http://www.cn-flora.ac.cn/) (Yao et al., 2018; Qin et al., 2022). The fruits of this plant have drawn extensive attention because of their abundant contents of diverse nutrients and bioactive substances, especially amino acids, vitamins, minerals, and polyphenols, particularly anthocyanins (Islam et al., 2017; Wang et al., 2018). In recent years, due to the growing pursuit of a healthy lifestyle, the medicinal value of *L. ruthenicum* has been increasingly acknowledged, and associated studies exploring its antioxidant, anti-inflammatory, and immune-regulating biological functions have been further deepened (Liu et al., 2020; Lu et al., 2020; Sharma et al., 2022). Additionally, as a pioneer plant with considerable salt, drought, cold tolerance, and sand fixation capabilities, *L. ruthenicum* plays an important role in desert management and ecological restoration in China (Islam et al., 2017).

Anthocyanins, which are important bioactive compounds found in *Lycium* *ruthenicum* fruits have strong antioxidant and anti-inflammatory functions (Hu et al., 2022). In addition, anthocyanins can enhance blood vessel elasticity, improve vision, and prevent cardiovascular diseases and cancer (Chen et al., 2022). These findings are not only crucial for the study of its medicinal value but also emphasize the importance of further research on the medicinal value of *L. ruthenicum*. In recent years, several flavonoid/anthocyanin biosynthesis structural genes and transcription factors (TFs) have been identified in *L. ruthenicum*, and their expression patterns have been analyzed (Xu et al., 2023; Zhao et al., 2021). However, the lack of high-quality whole-genome sequencing for *L. ruthenicum* has seriously impacted the exploration of anthocyanin formation, and the underlying genetic mechanisms remain largely unknown. In addition, anthracnose, root rot, gray spots, and powdery mildew severely affect the yield and quality of *Lycium* cultivation. Although several nucleotide-binding site (NBS) genes have been identified in the whole genomes of Solanaceae species including *Solanum tuberosum* (Jupe et al., 2012), *S*. *lycopersicum* (Shi et al., 2021), and *S*. *melongena* (Wei et al., 2020; Li et al., 2021; Jiang et al., 2023), the NBS gene family in *L. ruthenicum* has not yet been reported.

With the rapid development of third-generation sequencing technology, new technologies and ideas have emerged to advance the genomic research of medicinal and edible plants, and many medicinal plants have achieved gapless genomes, such as *Angelica sinensis* (Han et al., 2022), *Panax ginseng* (Song et al., 2024), *Penthorum chinense* (Wang et al., 2023b), *Vaccinium duclouxii* (Zeng et al., 2023), *Rhodomyrtus tomentosa* (Li et al., 2023)*, Malus domestica* (Su et al., 2024), and *Citrus reticulata* (Zhu et al., 2024); however, high-quality chromosome-scale genomes of *Lycium* *ruthenicum* have not been assembled until now.

By combining PacBio HiFi and ONT-ultra long (UL) via Hi-C, we have successfully assembled the nearly gapless reference genome of *Lycium* *ruthenicum*. This achievement marks the T2T reference genome of the *Lycium* genus, and lays a solid foundation for future studies. Based on the assembled genome, we not only further analyzed the genomic characteristics and conducted a comparative genomic analysis but also identified anthocyanin metabolic pathway genes and resistance-related genes through homology alignment. Subsequently, we analyzed their expression patterns integrated with the transcriptomic data. The high-quality reference genome of *L. ruthenicum* provides a valuable genetic resource for molecular breeding and genome evolution for the *Lycium* genus in the future.

## 2 Materials and methods

### 2.1 Plant materials and sequencing

Fresh leaves of wild *Lycium* *ruthenicum* were collected from Changji (44.30° N, 87.88° E) in the Xinjiang Uygur Autonomous Region, China. The fresh young leaves of samples were placed in a cryogenic vial with liquid nitrogen and stored at −80 °C. High-molecular-weight genomic DNA (gDNA) was extracted from young leaves using the DNeasy Plant Mini Kit (Qiagen) according to the manufacturer’s instructions. The quality and quantity of the extracted DNA were evaluated using agarose gel electrophoresis and a spectrophotometer.

For PacBio HiFi sequencing, two libraries were prepared from five or more micrograms (μg) of high-quality genomic DNA. The SMRTbell library was constructed using the SMRTbell Express Template Prep Kit 2.0 according to the following protocols. The library construction process began with DNA fragmentation, followed by end repair, adapter ligation, and size selection. To ensure the quality and purity of the libraries, they were purified to remove any contaminants and small fragments. The purified libraries were then sequenced on the PacBio Sequel II platform (Pacific Biosciences, CA, USA) according to the manufacturer’s instructions. Ultimately, the platform generated highly accurate high-fidelity HiFi data from two SMRT cells, totaling 164.85 Gb (Table S1).

Nanopore PromethION platform was used for Oxford Nanopore (ONT) sequencing. The library was constructed using the ONT genomic ligation sequencing kit SQK-LSK110 (Oxford Nanopore Technologies, UK) following standard protocols. Genomic DNA was fragmented using an ultrasonic crusher with precisely adjusted parameters to obtain fragments of appropriate lengths. Subsequently, adapter sequences provided by the kit were ligated to the ends of the fragmented DNA. After ligation under specific conditions, magnetic bead-based purification was performed to remove impurities and unligated DNA from the library. The raw data generated during the sequencing process was subjected to base-calling analysis using the Oxford Nanopore Guppy v.5.0.17 (Wick et al., 2019). The ONT sequencing data were then trimmed and filtered using NanoFilt v.2.3.0 (De Coster et al., 2018) to remove short (< 500 bp) and low-quality reads (< 7%), resulting in 73.45 Gb of clean data for further analysis.

For Hi-C sequencing, DNA was extracted from fresh leaves and fixed with paraformaldehyde to maintain the chromatin structure for 30 min. The fixed DNA was then digested with the restriction enzyme MboI, generating sticky-ended fragments. DNA polymerase was used to fill in these ends with specific deoxynucleotides, and biotin was added to the ends. Subsequently, DNA ligase ligated the fragments into chimeric circles. The ligated DNA underwent decrosslinking, followed by purification to remove contaminants. The purified DNA was sheared into 300–500 bp fragments to form the Hi-C library, which was sequenced on the Illumina NovaSeq 6000 platform with 150 bp paired-end reads to obtain Hi-C data for further analysis. The Hi-C data was filtered using Fastp v.0.23.2 (Chen, 2023). Finally, approximately 220 Gb of Hi-C clean data was generated (Table S2).

### 2.2 Genome assembly and quality assessment

PacBio HiFi reads were first used to estimate genome size and heterozygosity using Jellyfish v.2.2.7 (Marçais and Kingsford, 2011) and GenomeScope v.2.022 (Ranallo-Benavidez et al., 2020) with a 21-kmer. For *de novo* genome assembly, PacBio HiFi reads, ONT reads, and Hi-C data were assembled into contigs using Hifiasm v.0.19.5-r587 with the default parameters (Cheng et al., 2024). The BLAST approach was employed to compare and remove sequences with base-pair alignment > 80% that potentially originated from chloroplast or mitochondrial DNA of *Lycium* *ruthenicum* (Camacho et al., 2009). Subsequently, Hi-C reads were mapped onto the contig assembly genome of *L. ruthenicum* using Juicer v.1.7.6 (Durand et al., 2016a) with default parameters. Candidate chromosomes/scaffolds were generated after correcting misjoins and determining the order and orientation of the contigs through 3d-DNA pipeline (180419) (Dudchenko et al., 2017). Finally, the draft assembly was manually checked and refined using Juicebox v.1.11.08 (Durand et al., 2016b) resulting in the attainment of the final chromosome-scale genome.

For gap filling, NextDenovo v.2.3.0 (Hu et al., 2024) was employed for ONT long-read assembly. Gap filling was performed using PacBio HiFi reads, ONT reads, and ONT contigs with TGS-GapCloser v.1.2.1 (-x asm5) (Xu et al., 2020) and the GapFiller module in the quarTeT v.1.1.2 with default parameters (Lin et al., 2023). Centromeric regions were predicted using CentIER3 (Xu et al., 2024) with default parameters, and telomeric repeats (TTTAGGG) were predicted using the teloExplorer module in quarTeT v.1.1.2 (Lin et al., 2023) with default parameters. In addition, we counted the length, gene density, repeat sequence, and GC content of each chromosome with a window length of 50,000 bp, and visualized them using Circos v.0.69–8 (Gu et al., 2014).

Genome completeness and quality were evaluated using several methods. First PacBio HiFi reads were mapped using minimap2 v.2.17 (Li, 2018), and the mapping rate was subsequently calculated using SAMtools v.1.19 (Li et al., 2009). Additionally, the completeness of the genome was assessed using Benchmarking Universal Single-Copy Orthologs (BUSCO v.5.4) analysis based on the gene set from embryophyta_odb10 ortholog and eudicots_odb10 databases (Manni et al., 2021). The consensus quality (QV) was determined using Merqury v.1.3 (Rhie et al., 2020) with default parameters. The long terminal repeat (LTR) assembly index (LAI) was calculated using LTR_retriever v.2.9.0 (Ou et al., 2018).

### 2.3 Repeat sequence identification, gene prediction, and functional annotation

Genome annotation included repetitive sequence annotation, gene structure prediction, and gene function prediction. Initially, repeat sequences were predicted de novo using RepeatModeler v.2.0.2 (Flynn et al., 2020) and subsequently masked using RepeatMasker v.4.0 (Chen, 2004). The EDTA v.2.1.0 was employed to identify transposable elements (TEs) (Ou et al., 2019). A soft-masked genome was utilized for gene structure prediction.

For gene structure prediction, we employed the Braker3 pipeline (Gabriel et al., 2023). It automatically integrates RNA-seq and protein data. Initially, for RNA-seq prediction, RNA-seq reads from different tissues (roots, stems, leaves, flowers, and fruits; accession numbers SRR15037485, SRR12805573, SRR12805581, SRR12958754, SRR12958747, SRR23759243, SRR15037499) of *Lycium* *ruthenicum* were filtered using Fastp v.0.23.2 (Chen et al., 2023), and then mapped to the newly assembled genome using Hisat2 v.2.1.0 (Kim et al., 2019). Subsequently, we input the mapped RNA-seq data and protein data from *Vitis vinifera*, *Ipomoea triloba, Nicotiana tabacum, Solanum* *lycopersicum*, *S.* *tuberosum, Capsicum annuum*, *Lycium ferocissimum*, and *L. barbarum* into the automated process of Braker3. Finally, Braker3 integrated the two data types and predicted reliable genes using GeneMark-ETP v.4.65 (Brůna et al., 2020) and Augustus v.3.3.2 (Nachtweide and Stanke, 2019). The completeness of the annotation was assessed using BUSCO v.5.4 (Manni et al., 2021).

Functional annotation of the protein-coding genes was performed using eggNOG-mapper. Non-supervised orthologous group (NOG) assignments were made using the eggNOG database, along with Gene Ontology (GO) (Ashburner et al., 2000), Clusters of Orthologous Groups of proteins/euKaryotic Orthologous Groups (COG/KOG) (Koonin et al., 2004), and Kyoto Encyclopedia of Genes and Genomes (KEGG) (Kanehisa and Goto, 2000) databases for functional annotation.

### 2.4 Phylogenetic tree construction and evolution rate estimation

First, genome sequences and annotation files of 11 species were utilized to obtain the longest transcript sequence for each gene. OrthoFinder v.2.5.4 (Emms and Kelly, 2019) was employed to cluster orthologous, paralogous, and single-copy homologous genes of these 11 plants. For the phylogenetic analysis, single-copy orthologous genes of *L. ruthenicum* and 10 other representative plant species (*Amborella trichopoda*, *Arabidopsis thaliana*, *Vitis* *vinifera*, *Citrus sinensis*, *Ipomoea* *triloba*, *Nicotiana* *tabacum*, *Solanum* *tuberosum*, *S. lycopersicum*, *Lycium* *barbarum*, and *L. ferocissimum*) were aligned using MAFFT v.7.205 (Katoh and Standley, 2013), and ambiguously aligned regions were removed by applying trimAl v.2.0 (Capella-Gutierrez et al., 2009) with the parameter -gt 0.8. Subsequently, the maximum likelihood (ML) phylogenetic tree of the 11 species was constructed using RAxML v.8.2.13 with GTRGAMMA model (Stamatakis, 2014). Finally, the MCMCtree module of PAML v.4.7 (Yang, 2007) was used for divergence times estimation. Fossil calibration time was obtained from the TimeTree database (http://www.timetree.org). The time for comparison between the Solanaceae and Convolvulaceae was set at 75 million years ago (Mya), with the time range estimated to be [59.1–83.9 Mya]. Meanwhile, the differentiation time for *A. thaliana* and *C. sinensis* was set at 97 Mya [90.0–100.5 Mya]. The parameters for the MCMC tree program were configured as follows: the burn-in was set to 20,000,000, the sample frequency was set to 100, and the sample number was set to 5,000,000.

### 2.5 Gene family analysis

Gene family expansions and contractions were identified for each divergence node across the 11 species using CAFÉ v.5 (Han et al., 2013) with a *P*-value threshold of less than 0.05. Finally, the expanded and contracted gene families (*P* < 0.05) of *Lycium* *ruthenicum* were functionally annotated by enrichment with the GO and KEGG databases.

### 2.6 Gene duplication identification

Whole-genome duplication (WGD) events of *Vitis* *vinifera*, *Ipomoea* *triloba*, *Nicotiana* *tabacum*, *Solanum* *tuberosum*, *S. lycopersicum*, *Lycium* *barbarum*, *L. ferocissimum*, and *L. ruthenicum* were detected using MCscanX (Python version) (Wang et al., 2012), based on the gene-coding sequences and protein sequences. Additionally, *V. vinifera*, as a basal core eudicot lineage, has not undergone any additional WGD events following the ancient gamma duplication event shared by core eudicots. Therefore, we selected *V. vinifera* as a reference and conducted a synteny analysis between *V. vinifera* and *L. ruthenicum,* with at least five gene pairs required per syntenic block. Furthermore, DupGen_finder v.0.13.0 (Qiao et al., 2019) was used to identify duplicated genes in the *L. ruthenicum* genome with the default parameters, including WGD, tandem duplication (TD), transposed duplication (TRD), proximal duplication (PD), and dispersed duplication (DSD). KaKs_Calculator v.2 (Zhang, 2022) was employed to calculate the non-synonymous (*Ka*) and synonymous (*Ks*) values. Finally, we performed GO enrichment and KEGG functional analysis for duplicated genes using clusterProfiler v.4.0 (Wu et al., 2021) and visualized the results using the R package (Wang et al., 2010).

We calculated and compared the type and percentage of LTR retrotransposon replication in the genomes of *Lycium* *ruthenicum* with two Solanaceae species, *L. barbarum* and *L. ferocissimum*. Initially, we used LTRharvest v.1.5.11 (Ellinghaus et al., 2008) for de novo prediction of LTR retrotransposons. Subsequently, LTR_retriever v.2.9.0 (Ou and Jiang, 2018) was used to identify potential LTR retrotransposon sequences, and the insertion time (T = *Ks*/2μ; μ = 6.5e–9) was estimated based on the difference in synonymous nucleotide substitutions between the 5′-LTR and 3′-LTR of the same transposon, with a substitution rate of 6.5e–9 (Jiao et al., 2012).

### 2.7 Transcriptome analysis

RNA sequencing data of fruit samples at different developmental stages (H1: young stage, 9 days post-anthesis; H2: green stage, 15 days post-anthesis; H3: turning stage, 21 days post-anthesis; H4: red stage, 28 days post-anthesis; H5: ripe stage, 35 days post-anthesis) were utilized for transcriptome analysis, with three biological replicates for each sample (Table S14). The RNA sequencing data were obtained from a published paper (Cao et al., 2021). Raw data were filtered using Fastp v.0.23.2 (Chen, 2023) to trim adapters and low-quality bases. The clean reads were aligned to the *Lycium* *ruthenicum* reference genome using Hisat2 v.2.1.0 (Kim et al., 2019). The quantification of gene expression (Transcripts per Kilobase Million, TPM) was obtained using FeatureCounts v.2.0.1 (Liao et al., 2014). We set |log2 fold change| > 1 and an adjusted *p*-value < 0.05 as the criteria for differentially expressed genes (DEGs) between samples. The GO and KEGG enrichment analyses of DEGs were conducted using the R package clusterProfiler v.4.0 (Wu et al., 2021).

### 2.8 Identification of genes involved in anthocyanin biosynthesis

Genes related to the anthocyanin biosynthesis pathways (phenylalanine ammonia lyase: *PAL*; 4-coumarate CoA ligase: 4CL; cinnamate 4-hydroxylase: *C4H*; chalcone synthase: *CHS*; chalcone isomerase: *CHI*; flavanone 3-hydroxylase: *F3H*; flavonoid 3′-hydroxylase: *F3′H*; flavonoid 3′,5′- hydroxylase: *F3′5′H*; dihydroflavonol 4-reductase: *DFR*; anthocyanidin synthase: *ANS*; uridine diphosphate-glucose:flavonoid 3-O-glucosyltransferase: *UFGT*) in the *Lycium* *ruthenicum* genome (Song et al., 2022) were identified using the following criteria. Initially, the identification of the anthocyanin biosynthetic genes was carried out by the two-step BLAST method. We selected the publicly available protein sequences of anthocyanin biosynthesis-related genes in *Arabidopsis* *thaliana* as a reference and used Blastp v.2.10.0 (E-value < 1e–10, identity > 80%, score > 150) to identify anthocyanin biosynthetic genes in *L. ruthenicum*. Subsequently, all possible candidate genes were further identified by the NCBI Blastp process (E-value < 1e–5) (Camacho et al., 2009). After that, the accuracy of the genes was further verified by comparing proteins and conserved domains based on the Conserved Domain Database (CDD) and the Pfam database. For transcription factors (TFs), the protein sequence of the *L. ruthenicum* genome was submitted to plantTFDB (Jin et al., 2017) to identify the anthocyanin biosynthesis-related TFs with the best match in *A. thaliana*. Subsequently, the conserved domains of the identified TF protein sequences were verified following the same procedures as those for the anthocyanin biosynthetic genes, including comparing proteins and conserved domains based on the CDD and Pfam databases (MYB: PF00249; bZIP: PF00170; WD40: PF00400; bHLH: PF00010). Finally, the distribution of anthocyanin biosynthetic genes on chromosomes and the heatmap of gene expression were visualized using TBtools v.2.0 (Chen et al., 2020).

### 2.9 Resistance (R) gene identification

A conserved domain and homology search were conducted to identify the R genes in the genome of *Lycium* *ruthenicum*. HMMER v.3.1 (Potter et al., 2018) software with a hidden Markov model (HMM) profile was used to scan the NB-ARC (E-value < 1e–60) domain within the Pfam protein family (NB-ARC: PF00931). Finally, candidate genes harboring the NBS domain were validated using the NCBI CDD to ensure that they encoded the corresponding NBS candidate proteins. GO enrichment and KEGG functional annotation analysis for NBS genes were performed using the R package *clusterProfiler* v.4.0 (Wu et al., 2021). The heatmap of gene expression was visualized using TBtools v.2.0 (Chen et al., 2020).

## 3 Results

### 3.1 Genome sequencing and assembly

**Fig. 1:**
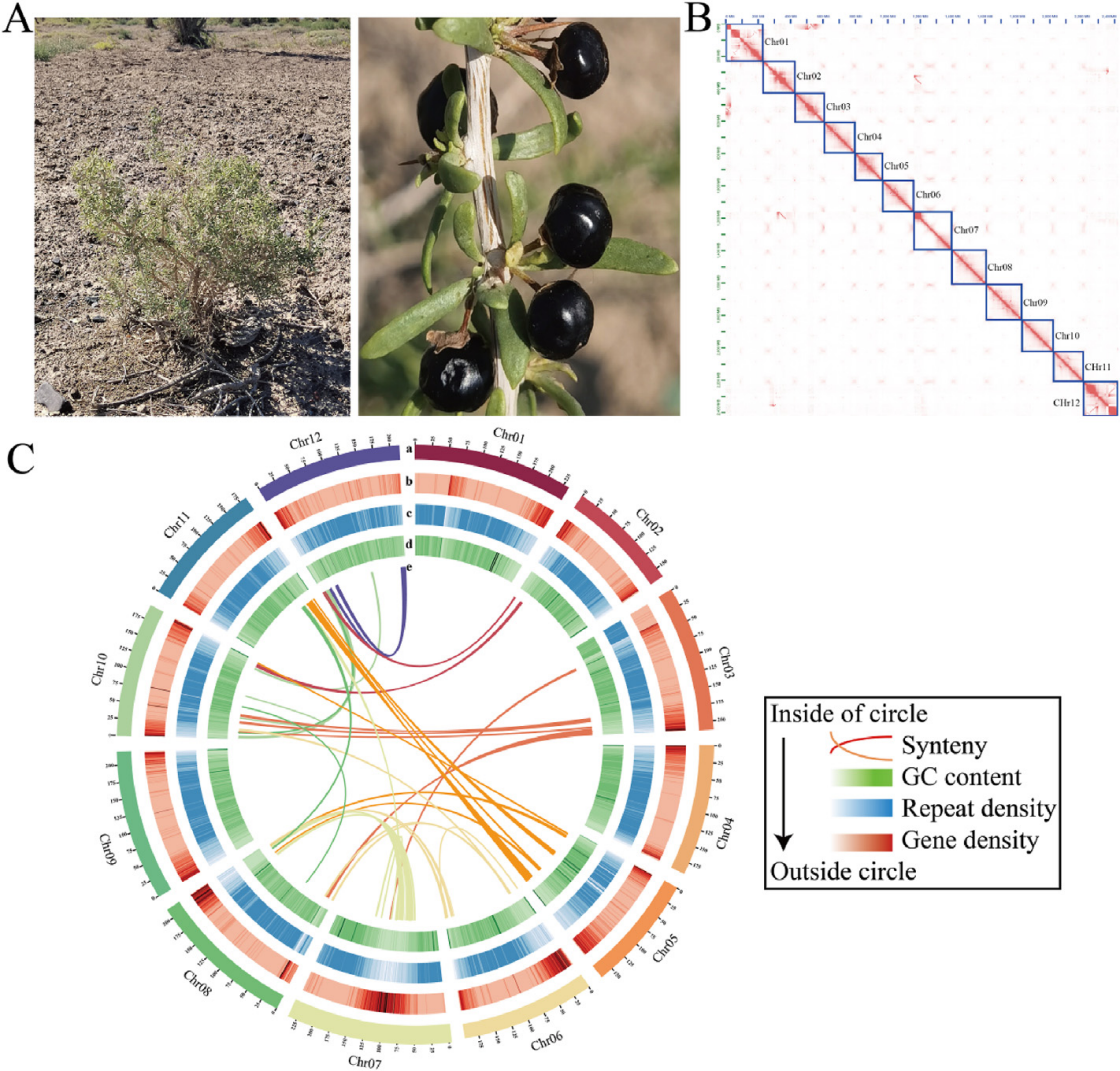
Overview of the *Lyciumruthenicum* genome. A. Image of *L. ruthenicum*. B. Hi-C interactions heat map of 12 chromosomes from *L. ruthenicum,* and individual chromosomes were represented by blue boxes. C. Circular plot showing basic genomic information of *L. ruthenicum* genome. (a): chromosome lengths, (b): gene density, (c): repeat sequence density, (d): GC content, and (e): the interior relationship between different chromosomes.

The estimated genome size of *Lycium* *ruthenicum* (Fig. 1A) was 2.26 Gb, with a heterozygosity of 0.998% heterozygosity using a 21-mer (Fig. S1). A total of 164.85 Gb HiFi reads, 73.45 Gb of clean ONT reads and 220 Gb Hi-C data were prepared for genome assembly (Tables S1–S2). We generated a preliminary genome assembly using Hifiasm v.0.19.5-r587, combined with HiFi reads, ONT data, and Hi-C data. After removing organellar sequences and redundant sequences, these reads were assembled into a 2.26 Gb genome containing 51 contigs, with a contig N50 value of 171.25 Mb. Furthermore, Hi-C data were mapped onto the assembled contigs, and 12 well-organized pseudochromosomes were successfully constructed (Fig. 1B), with a scaffold N50 size of 202.25 Mb, after excluding redundant sequences and short contigs (Table 1). Nine gaps were identified in the initially assembled genome; subsequently, ONT reads, ONT assembly contigs and HiFi reads were used to fill these gaps, resulting in the closure of five gaps. Finally, the complete genome of *L. ruthenicum* was constructed, with a size of 2.26 Gb and a GC content of 38.9% (Fig. 1C). The lengths of the 12 chromosomes ranged from 232.80 (Chr07) to 158.23 Mb (Chr02). There were four gaps in chromosomes Chr03, Chr04, Chr06, and Chr08, each being 500 bp length (Table S3). The genome assembly and chromosome length statistics are shown in Table 1 and Table S3.

**Table 1:** The *Lyciumruthenicum* genome assembly statistics.

Assembly feature	Statistics
**Scaffold**	
Total assembly size (bp)	2,422,165,955
Pseudochromosomes	12
Maximum contig length (bp)	233,546,130
Contig N50 length (bp)	212,077,492
Contig N90 length (bp)	168,456,903
Gap number	4
BUSCO completeness (%)	98.8
Number of protein-coding genes	38,882
Percentage of repeat sequences (%)	81.75

The BUSCO assessment results revealed that the assembled genome contained complete sets of core orthologous genes for embryophytes (98.5%) (Table S4) and eudicots (98.8%) (Table S4 and Fig. S2), with 93.3% being complete and single-copy genes. Moreover, the mapping rate of the HiFi reads to the genome achieved 99.88%, indicating high coverage of the assembly. The LTR assembly index (LAI) for *L. ruthenicum* genome was estimated to be 11.04, meeting the reference genome standards. The results of the K-mer statistical analysis showed that the QV value of the genome was 68.74, with each chromosome ranging from 68.08 to 72.22 (Table S5), and the genome consensus quality value was 85.81. These findings suggest that the assembled *L. ruthenicum* genome is complete and highly accurate.

Furthermore, the centromeric positions of the 12 chromosomes have been successfully predicted (Table S6). In addition, telomeres were detected at both ends of chromosomes Chr02 and Chr09 in the assembly, whereas they were found only at one end in the other nine chromosomes (Table S6). The results indicated that the genome assembled in this study is similar to that of the T2T standard.

### 3.2 Genome annotation

Repetitive sequence results show that 1,980,103,586 bp (81.75%) of the assembled genome sequences are occupied by repetitive regions. Retrotransposons were the most abundant, accounting for 73.01% of the genome, among which the LTR sequences of Gypsy and Copia superfamilies accounted for 43.14% and 8.41%, respectively (Table S7).

Additionally, by integrating RNA-Seq and homology-based approaches, we predicted 38,882 protein-coding genes, with an average gene length of 4308.14 bp. We functionally annotated 34,065 (87.61%), 34,065 (87.61%), 16,255 (41.81%), and 10,465 (26.92%) genes to the eggNOG, COG, GO, and KEGG databases, respectively. As a result, 37,149 genes (95.54% of the total) were annotated to at least one public database. Our annotation BUSCO analysis indicated a completeness of 98.2%, with 2285 complete BUSCOs and 1785 single-copy BUSCOs (Table S8).

### 3.3 Comparative genomics and gene family evolution analysis

**Fig. 2:**
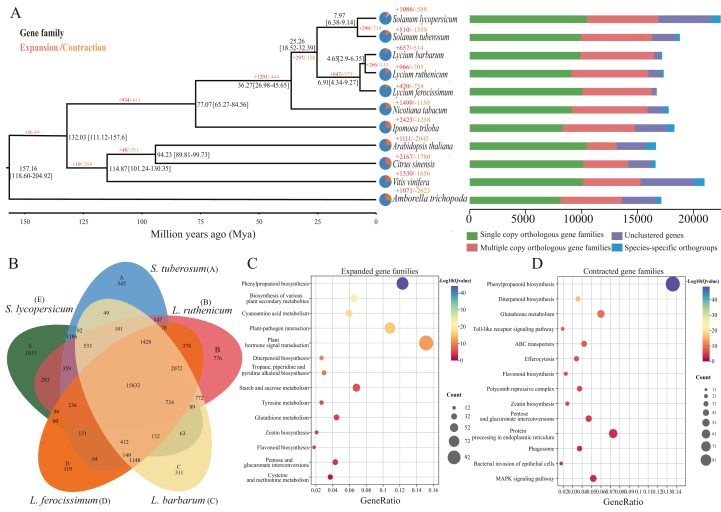
Phylogeny and gene family analyses of *Lyciumruthenicum*. A. Phylogenetic tree of *L. ruthenicum* and other ten plant species. The divergence time (million years ago, Mya) of each node was represented by a black number with the confidence range in brackets. The numbers of expanded, contracted gene families are shown in red and orange, while no changed gene families are shown in blue. Distribution of gene numbers and family sizes of 11 species (left). B. Gene family clustering diagram of five Solanoideae species (*Solanum tuberosum*, *S*. *lycopersicum*, *L. ruthenicum*, *L. barbarum*, *L. ferocissimum*). The letters in parentheses represent different species, and the numbers represent the number of common and unique gene families. C and D. KEGG enrichment for expanded (left) and contracted (right) gene families in *L. ruthenicum*.

To understand the evolutionary history of *Lycium* *ruthenicum*, the phylogenies of *L. ruthenicum* and 10 other published plant species were inferred using a concatenated dataset of 1978 single-copy genes (Fig. 2A). The ML tree results showed that *L. ruthenicum* formed a monophyletic group with its related species *L.* *barbarum* and *L.* *ferocissimum*. The divergence time between *L. ruthenicum* and *L. barbarum* is estimated to be 4.65 Mya [95% Highest Posterior Density (HPD): 2.90–6.35 Mya]. The most recent common ancestor (MRCA) of two *Lycium* species (*L. ruthenicum* and *L. barbarum*) split with *L. ferocissimum* occurred at about 25.26 Mya [95% HPD: 18.52–32.39 Mya].

Further investigation of the expansion or contraction of gene families across the 11 genomes revealed that a total of 26,182 gene families were shared among all 11 species, and 17,329 gene families were identified in the *L. ruthenicum* genome. A total of 350 gene families were significantly expanded (*P* < 0.05), while 209 were significantly contracted (*P* < 0.05; Fig. 2A) in the *L. ruthenicum* genome. These expanded genes were mainly related to phenylpropanoid biosynthesis, plant–pathogen interactions, cyanoamino acid metabolism, and various plant secondary metabolites (Fig. 2C). GO analysis showed that these genes were associated with sucrose transport, disaccharide transport, and oligosaccharide transport, indicating that expanded gene families may have played a vital role in the richness of secondary metabolites in the *L. ruthenicum* genome (Fig. S3A). In contrast, contracted genes were primarily related to phenylpropanoid biosynthesis, diterpenoid biosynthesis, the MAPK signaling pathway, and phagosome (Fig. 2D). GO enrichment analysis showed that these genes were associated with cellular responses to cold, response to hydrogen peroxide, and calcium-release channel activity (Fig. S3B).

To understand the genetic characteristics of the *Lycium* *ruthenicum* genome compared with Solanoideae species, we selected five Solanoideae species from the 11 species to identify species-specific gene families (Fig. 2B). The results showed that 15,832 gene families were shared among the five Solanoideae species, while 776 gene families were unique to *L. ruthenicum*, which was more than those of *L. barbarum* (456), *L. ferocissimum* (657), and *S*. *tuberosum* (231) (Tables S9–S13). We further conducted GO enrichment analysis on the five Solanoideae species and compared them with *L. ruthenicum*. The GO results indicated that the unique gene families of *L. ruthenicum* were significantly enriched in functional categories related to defense response, salt stress, water deprivation, and oxidative stress. These species-specific gene families may be related to the environmental adaptability of *L. ruthenicum* (Table S13).

### 3.4 Whole-genome duplication (WGD) events

**Fig. 3:**
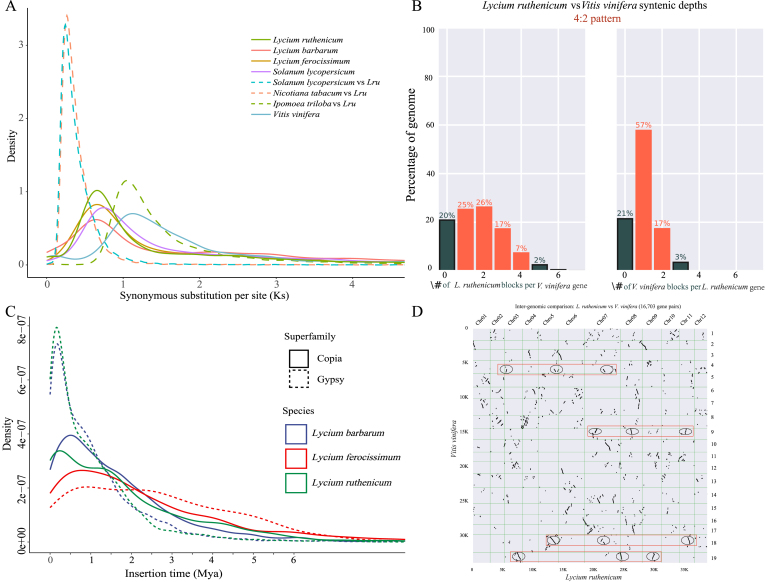
Genome evolution of *Lyciumruthenicum.* A. *Ks* distributions of paralogs and orthologous genes in the genomes of *Ipomoea triloba*, *Vitis vinifera*, *Nicotiana tabacum*, *Solanumlycopersicum*, *L. barbarum*, *L. ferocissimum* and *L. ruthenicum* (*Lru*). B. Syntenic depth analysis between *L. ruthenicum* and *V. vinifera*. C. Insertion time of LTRs in *L. ruthenicum, L. barbarum*, and *L. ferocissimum*; Mya: Million years ago. D. Homologous dot plot between *L. ruthenicum* and *V. vinifera.* Collinear blocks between the *L. ruthenicum* and *V. vinifera* chromosomes are highlighted by the red solid box.

To confirm the WGD events in *Lycium* *ruthenicum*, we selected six species (*Vitis* *vinifera, Ipomoea* *triloba*, *Nicotiana* *tabacum*, *Solanum* *lycopersicum*, *L. barbarum*, and *L. ferocissimum*) to calculate the *Ks* values for homologous genes between two species or within a single species (Fig. 3A). Our analysis found prominent *Ks* peak at 0.66 for *L. ruthenicum*. The *Ks* values of orthologous gene pairs of *L*. *ruthenicum* and *I. triloba* from the Convolvulaceae family showed a significant peak at *Ks* = 1.2. The five Solanaceae species showed one signature *Ks* peak at approximately 0.66–0.75. These results indicate that Solanaceae species may share a WGD event that occurred after divergence from the Convolvulaceae family. The nearly 4:2 orthologs ratio (Fig. 3B), and the 16,703 colinear gene pairs (Fig. 3D) detected between *L. ruthenicum* and *V. vinifera* indicate that WGD events occurred within the Solanaceae family. Furthermore, no species-specific whole-genome duplication events were detected in *L. ruthenicum*, as shown in Fig. S4.

The percentage of LTR retrotransposon replication in *L. ruthenicum* genome (LTRs: 73.01%, Gypsy/DIRS1: 43.14%) was higher than those in *L. barbarum* (LTRs: 66.73%, Gypsy/DIRS1: 37.61%) and *L. ferocissimum* (LTRs: 44.70%, Gypsy/DIRS1: 18.06%) (Fig. 3C), and a large number of insertions of LTRs initiated roughly ∼3.0 Mya (Fig. 3C).

### 3.5 Duplicated gene analyses

**Fig. 4:**
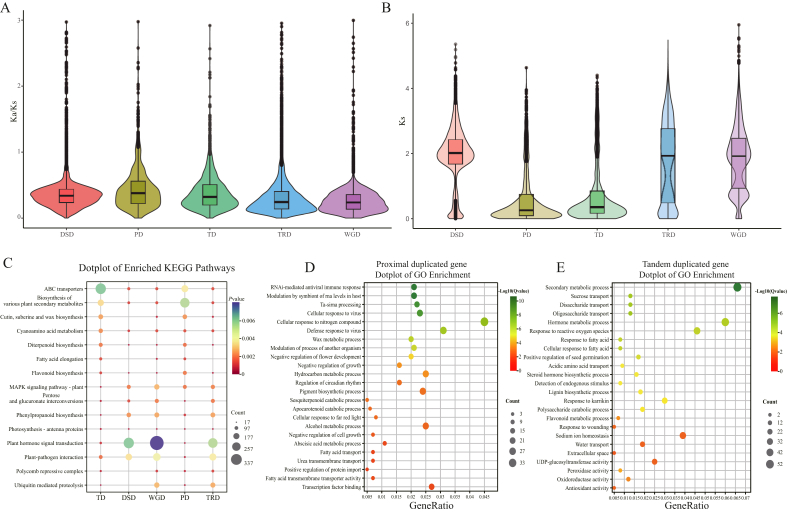
Duplicated gene in *Lyciumruthenicum* genome. A and B, *Ka*/*Ks* ratio and value of five types duplicated gene. C, KEGG and D, GO enrichment for five different types duplicated genes (*P* < 0.05). DSD: dispersed duplication, PD: proximal duplication, TD: tandem duplication, TRD: transposed duplication, and WGD: whole-genome duplication.

We identified 26,015 duplicated genes, which were classified into five distinct categories: 10,668 (41.01%) WGD, 6504 (25.00%) dispersed duplication (DSD), 6121 (42.50%) transposed duplication (TRD), 1775 (3.0%) tandem duplication (TD), and 947 (3.64%) proximal duplication (PD). We compared the *Ks* and *Ka*/*Ks* ratios among the five different duplications, revealing that PD tended to have higher *Ka*/*Ks* ratios (Fig. 4A) and lower *Ks* values (Fig. 4B). These results suggest that PD duplicate genes have undergone a more relaxed purifying selection.

Furthermore, we performed GO and KEGG analyses of the different types of duplicated genes. The KEGG enrichment analysis showed that PD was enriched in the biosynthesis of various plant secondary metabolites and flavonoids (Fig. 4C). TD gene sets were enriched in the biosynthesis of various metabolites such as phenylpropanoids, flavonoids, pese and glucuronate interconversions, and MAPK signaling pathways (Fig. 4C). WGD was enriched in plant hormone signal transduction, pathogen interaction, the MAPK signaling pathway, and phenylpropanoid biosynthesis (Fig. 4C). DSD was mainly enriched in plant hormone signal transduction, pathogen interaction, and phenylpropanoid biosynthesis (Fig. 4C). GO enrichment analysis showed that these duplicated genes were related to phospholipase activity, organophosphate catabolic processes, nucleoside phosphate catabolic processes, fruit dehiscence, and secondary metabolic processes (Fig. 4D and E). Overall, these results indicate that different types of gene duplications enriched biosynthetic genes related to important metabolites, cell signal transduction, and growth regulation in *Lycium* *ruthenicum* (Fig. S5).

### 3.6 Exploration of key genes in the anthocyanin biosynthesis accumulation

**Fig. 5:**
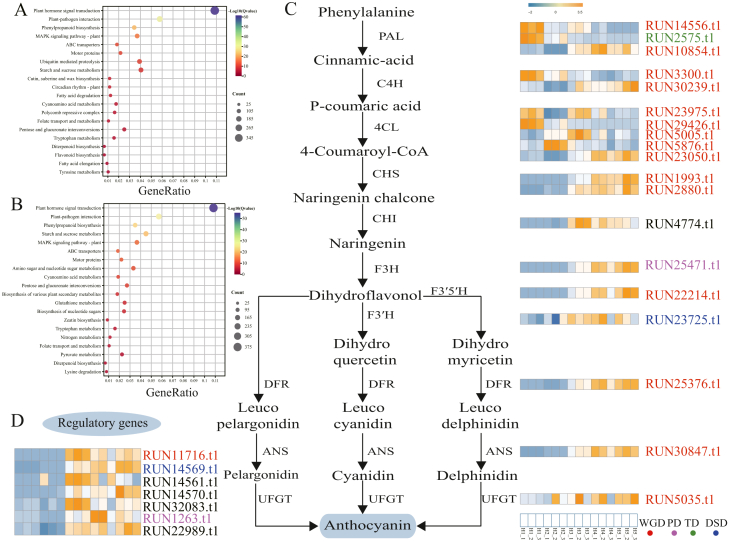
Anthocyanin biosynthesis in *Lyciumruthenicum* fruit. A and B. KEGG enrichment in upregulated and downregulated genes in *L. ruthenicum* fruit. C. Heatmap showing the differential expression of anthocyanin biosynthesis genes according to the transcriptome data of *L. ruthenicum* fruit. Different colors are used to represent different types of duplication genes. DSD: dispersed duplication, PD: proximal duplication, TD: tandem duplication, and WGD: whole-genome duplication. D. Heatmap showing the expression of transcription factor according to the transcriptome data of *L. ruthenicum* fruit.

We reanalyzed the transcriptome sequencing data for fruit developmental stages based on our newly assembled genome (Table S14). A total of 16,060 genes (from pairwise comparisons) were identified as differentially expressed genes (DEGs; |log2 fold change| >1 and *P*_adj_ < 0.05) during fruit development. Among these, 7384 genes were up-regulated, and 8676 genes were down-regulated in the fruits. The KEGG pathway enrichment analysis results of these DEGs are shown in Fig. 5A and B. Among these pathways, plant hormone signal transduction, plant–pathogen interaction, phenylpropanoid biosynthesis, fatty acid biosynthesis, carotenoid biosynthesis, fatty acid degradation, glycolysis/gluconeogenesis, flavonoid biosynthesis, and glycolipid metabolism played important roles in the fruit development of *Lycium* *ruthenicum*.

The most notable feature of *Lycium* *ruthenicum* is its high anthocyanin content in fruit. Although the anthocyanin biosynthesis pathway has been extensively studied in *L. ruthenicum* over the past few years, we have gained new insights into anthocyanin biosynthesis by combining high-quality genomic and transcriptome analyses. Based on the *L. ruthenicum* T2T genome, we identified 19 DEGs, candidate enzymatic genes in the anthocyanin biosynthesis pathway, including three *PAL*, two *C4H*, five 4CL, two *CHS*, one *CHI*, one *F3′H*, one *F3′5′H*, one *DFR*, one *ANS*, and one *UFGT* gene. KEGG enrichment analysis of DEGs revealed that these 19 candidate genes of *L. ruthenicum* were mainly enriched in the phenylpropanoid biosynthesis and flavonoid biosynthesis pathways. Further, the expression of these genes in the fruit was calculated using the RNA-Seq data (Fig. 5B). Five genes, *RUN14556.t1* (*PAL*)*, RUN2575.t1* (*PAL*)*, RUN3300.t1* (*C4H*)*, RUN23975.t1* (4CL) and *RUN29426.t1* (4CL)*,* were highly expressed in the early stages (H1 and H2) of *L. ruthenicum* fruit growth and decreased gradually, whereas the expression of the other 12 structural genes was quite low in the early stage and progressively increased in later stages (H3–H5) (Fig. 5C). These 19 genes are involved in all three stages of anthocyanin synthesis, and differences in their expression levels may have significant effects on anthocyanin synthesis in *L. ruthenicum* berries.

These genes were primarily concentrated on chromosomes Chr01, Chr03, Chr04, Chr06, Chr07, Chr08, Chr09, Chr10, and Chr11 (Fig. S6). Genes *CHI*, *F3H*, *F3′H*, *F3′5′H*, *DFR*, *ANS,* and *UFGT* are present as single copies, whereas all other genes have multiple copies. Among these structural genes, 15 are associated with WGD, one with DSD, and one with TD, indicating that different types of duplicated genes play crucial roles in the anthocyanin biosynthetic pathway in *Lycium* *ruthenicum* (Fig. 5C).

In addition, we identified seven TFs involved in the anthocyanin biosynthesis pathway in *Lycium* *ruthenicum* fruit, including four MYB genes (four MYB13*: RUN14561.t1*, *RUN11716.t1*, *RUN14569.t1*, *RUN14570.t1*), one bHLH gene (*RUN1263.t1*), one bZIP gene (*RUN32083.t1*), and one WD40 gene (*RUN22989.t1*). The expression of these seven TFs was higher in the later stages (H3–H5) of *L. ruthenicum* fruit growth (H3–H5), suggesting that these TFs may be involved in the regulation of anthocyanin synthesis in *L. ruthenicum* berries (Fig. 5D).

### 3.7 Resistance (R) gene identification

Resistance (R) genes play a crucial role in the process by which plants resist invasion by external pathogens. The identification of R genes can accelerate the process of identifying candidate disease-resistance genes (Jupe et al., 2012). In this study, based on domain architectures, 154 candidate NBS (nucleotide-binding site) genes were identified and classified into six subfamilies in the *L. ruthenicum* genome, including Coiled-Coil (CC)-NBS-leucine-rich repeat (LRR) (56), Toll interleukin-1 receptor (Tir)-NBS-LRR (31), CC-NBS (22), Tir-NBS (7), NBS-LRR (22), NBS (16) (Table S15). GO enrichment analysis of the NBS genes indicated that these genes were primarily related to the hypersensitive response, programmed cell death induced by symbionts, and innate immune response (Fig. S7A), whereas KEGG enrichment was related to plant–pathogen interactions and the MAPK signaling pathway (*P* < 0.05) (Fig. S7B).

The greatest number of NBS genes was found on Chr09 and Chr06, the lowest number was found on Chr05, and most NBS genes were clustered at the distal end of the chromosome (Fig. S8). Among the 154 NBS genes, the majority were derived from WGD (56); 24 belonged to proximal duplication, 35 to tandem duplication, and 8 to dispersal duplication.

**Fig. 6:**
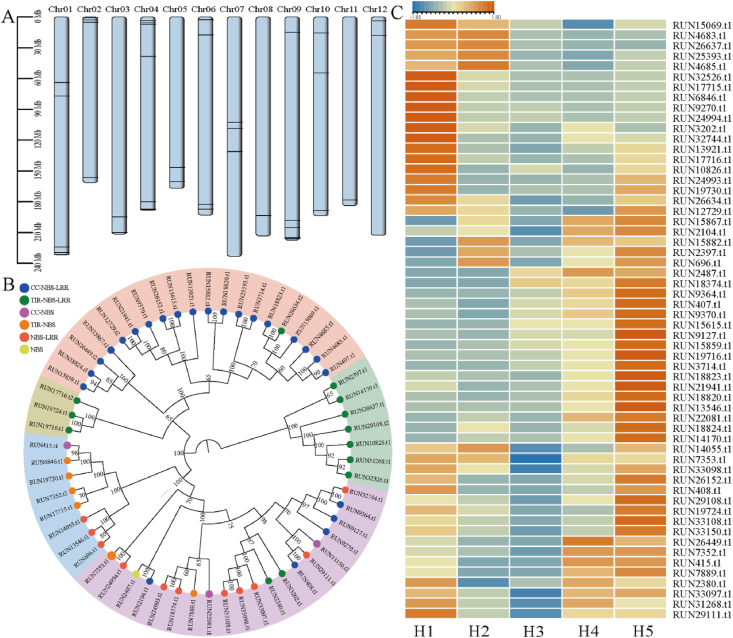
Phylogenetic and transcriptome analyses of NBS (nucleotide-binding site) genes in the *Lyciumruthenicum* fruit. A. Clustered distribution of 58 NBS genes on chromosomes, with gene locations marked in black. B. Phylogenetic tree based on 58 NBS disease-resistant protein amino acid sequence in *L. ruthenicum* fruit. CC-NBS-LRR: Coiled-Coil (CC)-NBS-leucine-rich repeat (LRR); Tir-NBS-LRR: Toll interleukin-1 receptor (TIR)-NBS-LRR. C. The expression of 58 NBS disease-resistant genes in the *L. ruthenicum* fruit.

To further understand the phylogenetic relationship of the NBS genes and their expression patterns during fruit development, we constructed a phylogenetic tree and performed DEGs analysis among the H1–H5 fruit samples (Table S16). A total of 58 differentially expressed candidate NBS genes were identified. Phylogenetic analysis showed that these genes are closely related (Fig. 6B) and are distributed in clusters at the ends of chromosomes (Fig. 6A). The expression patterns revealed that more than half of the genes were highly expressed during the H1 and H5 stages (Fig. 6C). This suggests that these genes play an important role in the development of resistance during fruit development.

## 4 Discussion

The assembly of the T2T genomes of medicinal plants is crucial for exploring genome evolution and identifying key genes related to important traits (Wang et al., 2023a; Yang et al., 2024). *Lycium* *ruthenicum* is a well-known plant with medicinal, economic, and ecological value, and its genome is of great significance for the development of new drugs and the understanding of pharmacological mechanisms. Previous studies utilized Illumina short-reads and PacBio technologies to assemble a scaffold-level genome of *L. ruthenicum* (Cao et al., 2021). However, due to the obvious fragmentation and low continuity in the assembly results, no high-quality genomic data have been published to date (last accessed on 4 October 2024). PacBio HiFi reads are highly accurate, and ONT reads have long read lengths; both have been successfully applied in complex genome assemblies (Feng et al., 2024). To better utilize the genomic data to support studies on the evolution and molecular breeding of *L. ruthenicum,* we assembled the high-quality, 2.26 Gb, nearly gap-free *L. ruthenicum* reference genome by integrating PacBio HiFi, ONT, and Hi-C technologies. The newly assembled genome comprises 12 chromosomes, with a contig N50 of 171.25 Mb, a scaffold N50 of 202.25 Mb, and 99.88% coverage of the full genome (Figs. 1C and S2). The quality of this assembly was markedly improved compared to the previously published genome of *L. ruthenicum* (contig N50 of 16.14 Mb and a scaffold N50 of 155.39 Mb) (Cao et al., 2021). Additionally, the BUSCO assessment value (98.8%) suggests that the newly assembled genome is superior to the previously published genome (Cao et al., 2021). These results indicate that the quality of assembly and continuity were markedly improved compared to those of the recently published *L. ruthenicum*, *L.* *barbarum* (Cao et al., 2021), and *L*. *chinense* (Yang et al., 2023). Therefore, our results indicate the establishment of the T2T genome of the *Lycium* genus, which lays a solid foundation for future genomic research and the breeding of new varieties.

Whole gene duplication and transposable element insertion are the main factors that increase genome size (Grover and Wendel, 2010). In this study, the assembled genome size of *Lycium* *ruthenicum* was larger than those of *L. barbarum* (1.77 Gb), *L. chinense* (1.538 Gb), and *L.* *ferocissimum* (1.15 Gb). Our analysis of WGD events indicated that Solanaceae species experience a new WGD event (*Ks* peak at 0.66–0.75) in addition to the ancient WGD event shared in eudicots, which is also believed to have occurred in most Solanaceae species, *Solanum* *melongena* (Wei et al., 2020), *L*. *barbarum* (Cao et al., 2021), and *Solanum muricatum* (Song et al., 2022). However, no species-specific whole-genome duplication events were detected in *L. ruthenicum* (Fig. S4). Notably, the results of the repeat sequence analysis indicated that the ratio of LTRs was higher in *L. ruthenicum* (73.01%) than in *L. barbarum* (66.73%) or *L. ferocissimum* (44.70%). Therefore, we inferred that the larger genome size of *L. ruthenicum* is likely due to the insertion of LTR elements, particularly Gypsy/DIRS1. Similar LTRs expansion events have been reported in the genomes of *Dendrobium officinale* (Niu et al., 2021) and *Ammopiptanthus mongolicus* (Feng et al., 2024).

LTRs are the most important repeat sequences in the genome, and their insertion at new locations can generate novel genes and phenotypic variations, aiding the adaptive evolution of species (Stapley et al., 2015; Niu et al., 2019). In the present study, the time of LTR insertion revealed that the Gypsy/DIRS1 insertion in the *Lycium* *ruthenicum* genome mainly occurred after approximately 3.0 Mya, especially during the Quaternary (Fig. 3C). This timeframe coincided with the continuous uplift of the Qinghai-Tibet Plateau, the rapid uplift of the Tianshan Mountains (2.6 Mya), and the subsequent aridification of the climate in northwest China (Meng et al., 2015; Liu et al., 2014). Therefore, we infer that the insertion of LTRs may have promoted the genetic diversity of *L. ruthenicum* and facilitated its rapid adaptation to the arid environment of Northwest China. Further studies are required to gain a comprehensive understanding of the role of LTRs in the evolutionary patterns of the genome in this species.

Species-specific gene families, which are crucial for diverse biological and metabolic processes in plants, significantly influence their unique traits and adaptations (Harris and Hofmann, 2015; Fu et al., 2022; Yu et al., 2022). Our analysis of species-specific gene families revealed a significant association with biological processes essential for survival under stress conditions, such as defense mechanisms, tolerance to salt, water scarcity, and oxidative stress (Table S13). These findings imply that the unique genetic makeup of *L. ruthenicum* endows it with a superior capacity to withstand drought compared to other species, such as *Solanum* *tuberosum*, *S.* *lycopersicum*, *Lycium* *barbarum*, and *L. ferocissimum* (Tables S9–S13). Thus, we infer that these species-specific gene families may play a key role in enhancing the resilience and adaptability of *L. ruthenicum* under harsh arid conditions.

The most notable feature of *Lycium* *ruthenicum* is its high anthocyanin content in fruit (Yao et al., 2018; Qin et al., 2022). High-quality genomes of *L. ruthenicum* are crucial for identifying and elucidating the genes involved in the anthocyanin/flavonol biosynthesis pathway. Here, we identified 19 enzymatic genes and seven TFs involved in the anthocyanin biosynthesis pathway in *L. ruthenicum* berries, with most genes showing high expression during the fruit ripening stages (H3–H5) (Fig. 5C). We inferred that these enzyme genes may directly promote the efficiency of anthocyanin synthesis, whereas TFs may regulate the transcription of structural genes to further affect anthocyanin synthesis (Fig. 5C); however, enzymatic activity cannot be ruled out. Future functional validation of these genes may help elucidate the mechanisms underlying anthocyanin synthesis. Furthermore, the presence of multiple copies of several structural genes suggested that various gene duplication events (WGD, TD, PD, and DSD) contributed to an increase in the number of biosynthesis-associated genes (Fig. 5C). Similar results have been reported for *Angelica* *sinensis* (Han et al., 2022), *Panax* *ginseng* (Song et al., 2024), and *Rhododendron simsii* (Yang et al., 2020). Overall, the identification of these structural genes and TFs offers new insights into the anthocyanin synthesis pathways during the fruit ripening process of *L. ruthenicum.*

Additionally, the exploration of resistance genes in *Lycium* *ruthenicum* is helpful for improving its resistance and for genetic breeding of *L. ruthenicum* (Dangl and Jones, 2001; McHale et al., 2006; Shao et al., 2019). We identified 154 NBS genes, most of which were highly expressed in the early and ripe stages of fruit development, suggesting that NBS-LRR genes play important roles in fruit pathogen resistance in *L. ruthenicum* (Fig. 6C). Different types of gene duplications, including WGD, TD, PD, and DSD, likely contributed to an increase in the number of NBS gene families, providing candidate genes for further exploration of the resistance gene family within the *Lycium* genus.

## 5 Conclusion

We assembled a nearly gapless T2T genome of *Lycium* *ruthenicum*, revealed its evolutionary patterns, and identified candidate genes related to anthocyanin synthesis and resistance. These research achievements not only enrich our understanding of the *Lycium* genome but also provide valuable genetic resources for important agronomic traits.

## CRediT authorship contribution statement

**Gulbar Yisilam:** Writing – review & editing, Writing – original draft, Software, Methodology, Formal analysis, Data curation. **Enting Zheng:** Writing – review & editing, Software, Formal analysis, Data curation. **Chuanning Li:** Writing – review & editing, Software, Formal analysis, Data curation. **Zhiyong Zhang:** Writing – review & editing, Visualization, Methodology, Conceptualization. **Ying Su:** Writing – review & editing, Validation, Data curation. **Zhenzhou Chu:** Writing – review & editing, Validation, Data curation. **Pan Li:** Writing – review & editing, Writing – original draft, Visualization, Supervision, Resources, Conceptualization. **Xinmin Tian:** Writing – review & editing, Writing – original draft, Visualization, Validation, Supervision, Resources, Project administration, Methodology, Investigation, Funding acquisition, Conceptualization.

## Data availability

The genome assembly, HiFi, Hi-C and ONT reads data have been deposited into the National Center for Biotechnology Information Sequence Read Archive database with project numbers: PRJNA1201566, PRJNA1201640 and PRJNA1201639. The genome assembly data is also available on the Figshare platform (https://doi.org/10.6084/m9.figshare.28156994).

## Declaration of competing interest

The authors declare that they have no known competing financial interests or personal relationships that could have appeared to influence the work reported in this paper.
