# Supplementary material for: The chromosome-scale genome of black wolfberry (Lycium ruthenicum) provides useful genomic resources for identifying genes related to anthocyanin biosynthesis and disease resistance
Source: Plant Divers. 2025 Jan 6;47(2):201–13. doi: 10.1016/j.pld.2025.01.001 (PMC11963026; doi:10.1016/j.pld.2025.01.001)
Supplement: Multimedia component 2 [file mmc2.docx]

**Supplemental Figures**


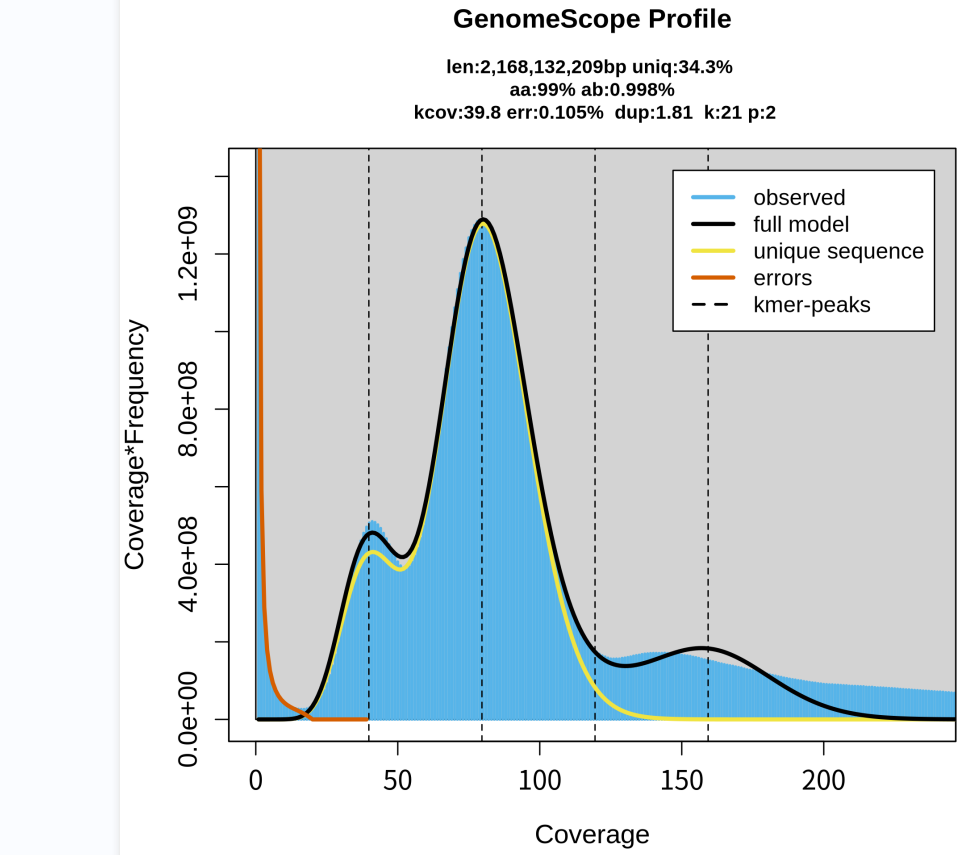


**Figure S1. The evaluation of *L. ruthenicum* genome size.** Genome scope profiles of 21-mer analysis. The X-axis represented the k-mer depth and the Y-axis represented the frequency of the k-mer for a given depth.


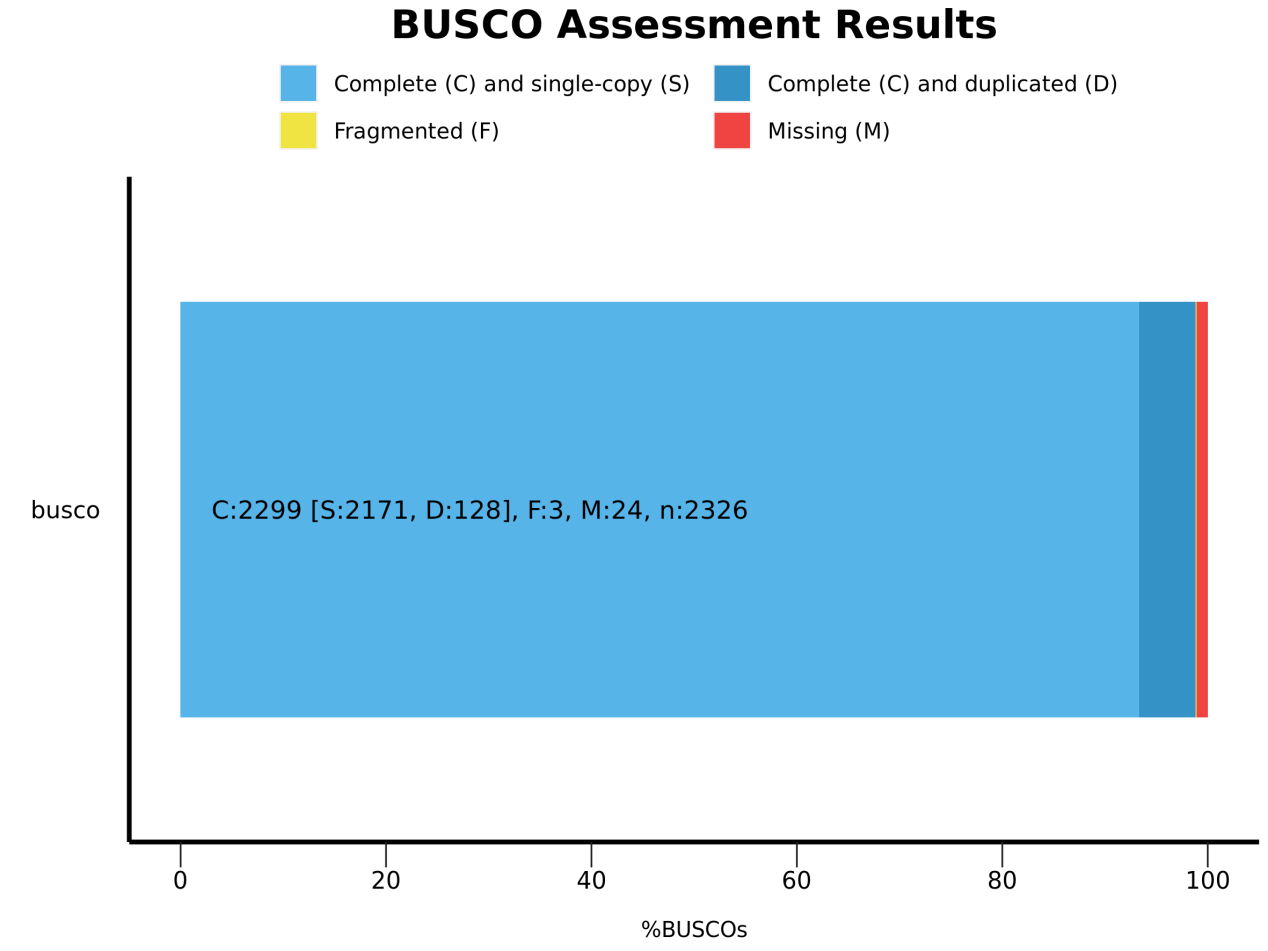


**Figure S2. BUSCO scores of the assembled genomes of *L. ruthenicum***


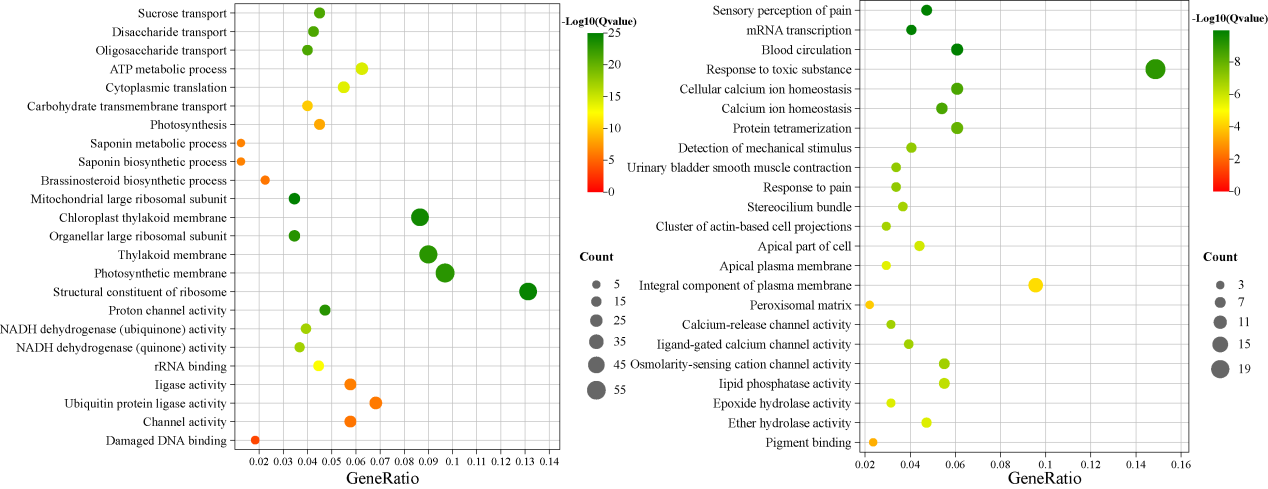


**Figure S3. The GO enrichment analysis for expanded (left) and contracted (right) gene families in** ***L. ruthenicum.*** The enriched terms with adjusted *P* < 0.05 are presented. Color of the bubbles indicates statistical significance of the enriched terms; size of the bubbles indicates number of genes.


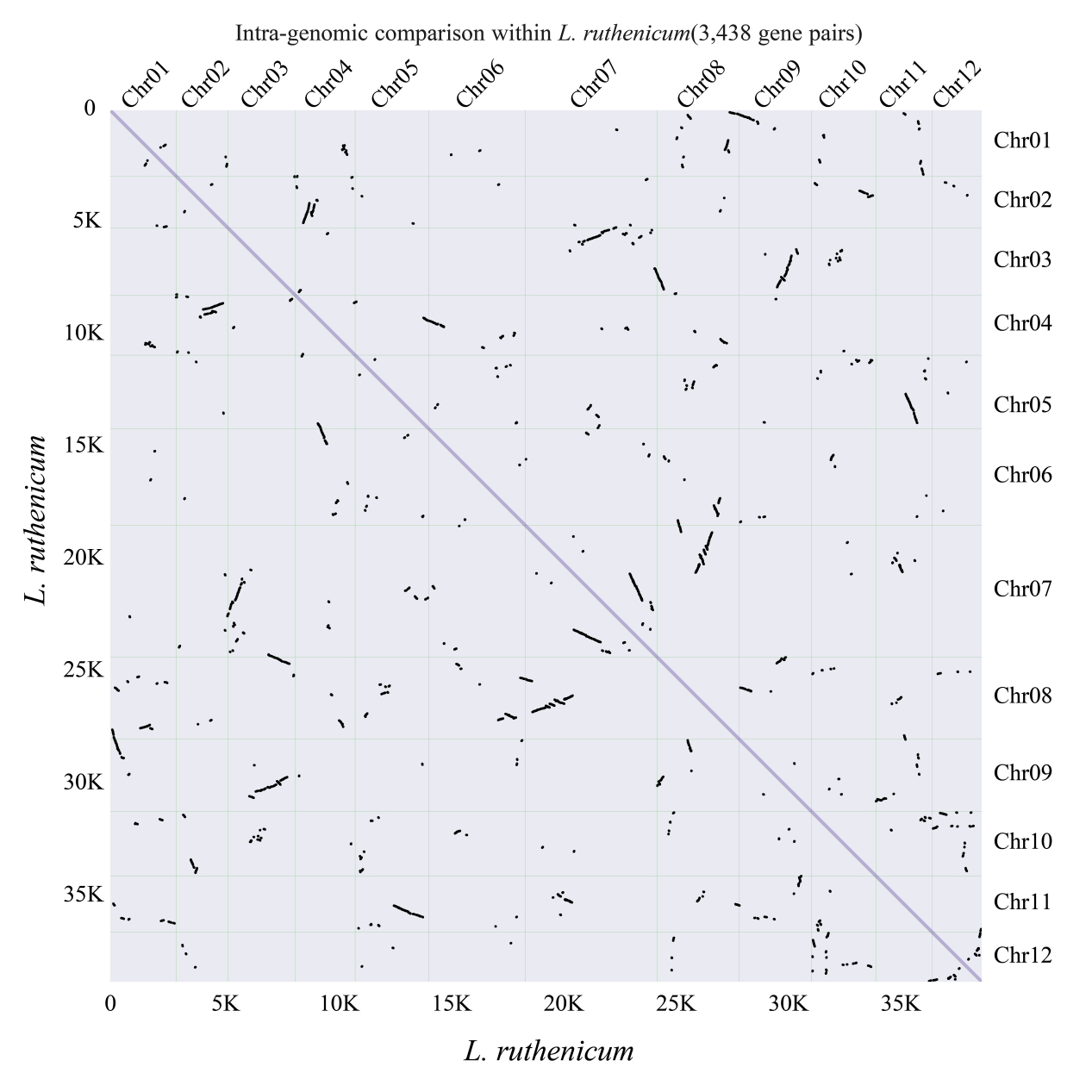


**Figure S4. Dot plots analyses for *L. ruthenicum* genome**


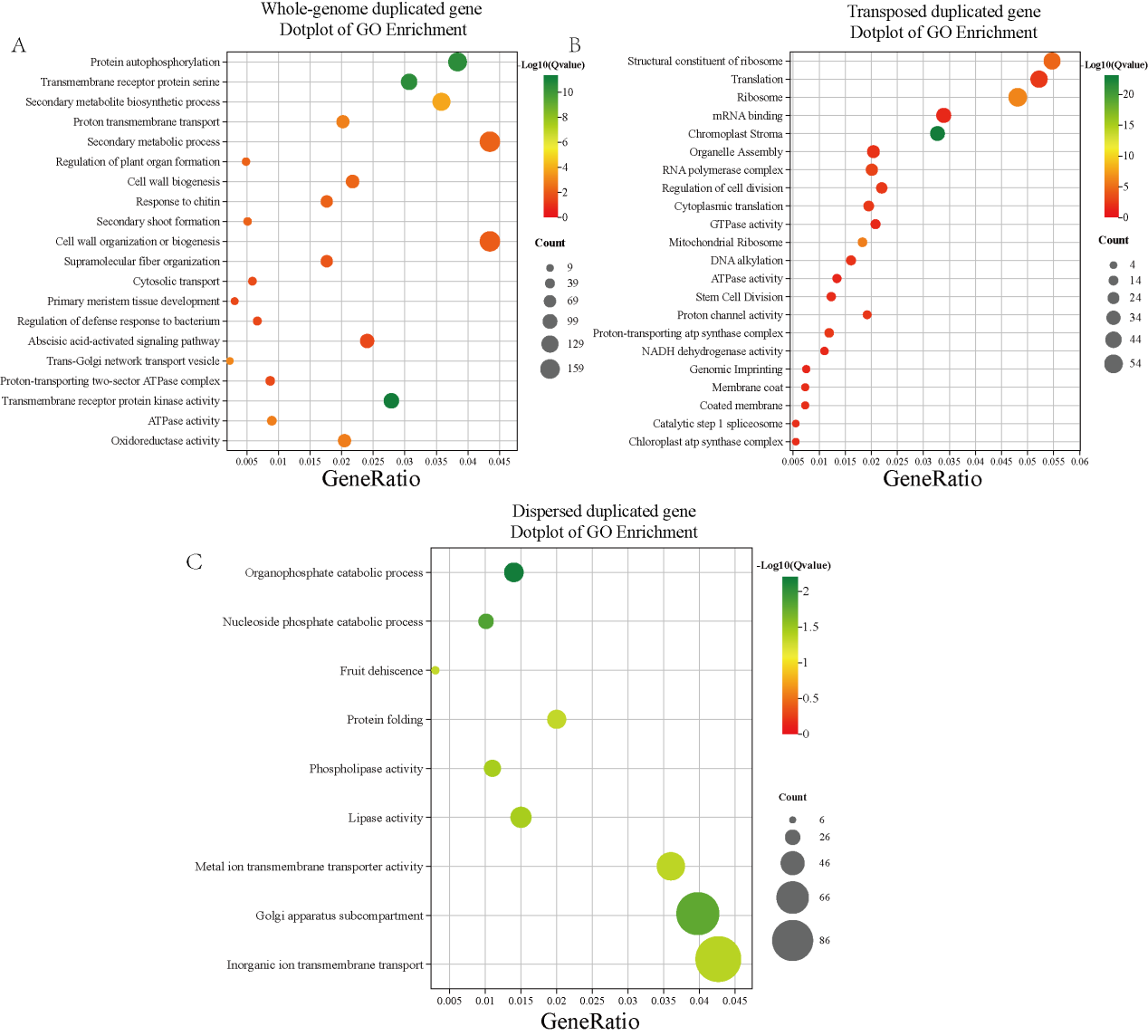


**Figure S5. Gene ontology (GO) enrichment analyses of genes from different types of gene duplication.** The enriched terms with adjusted *P* < 0.05 are presented. Color of the bubbles indicates statistical significance of the enriched terms; size of the bubbles indicates number of genes.


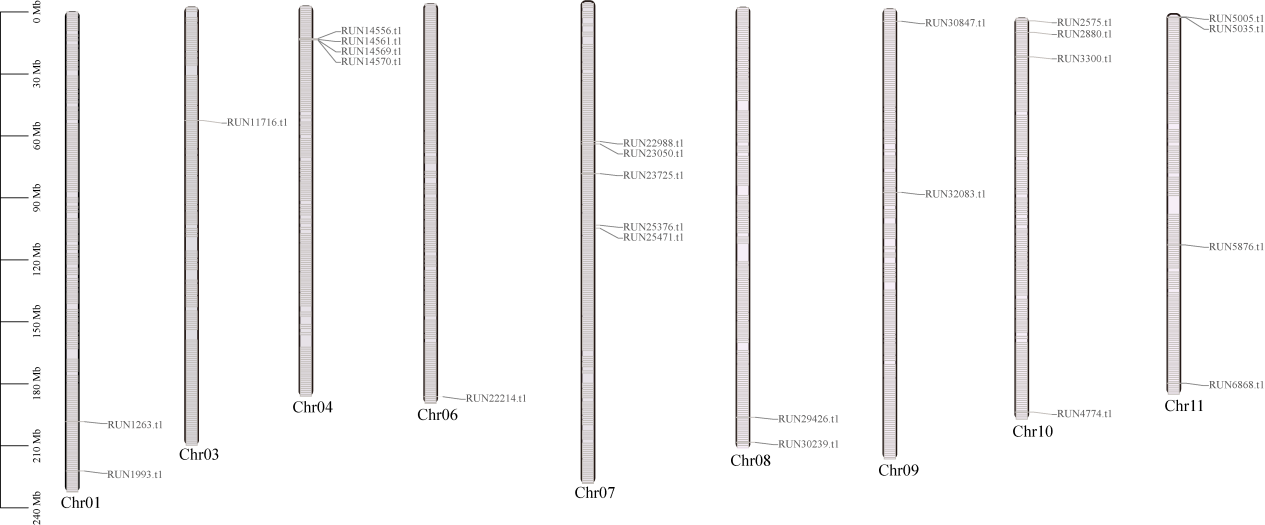


**Figure S6.** Location of predicted anthocyanin biosynthesis-related structure genes and transcription factors genes on chromosomes


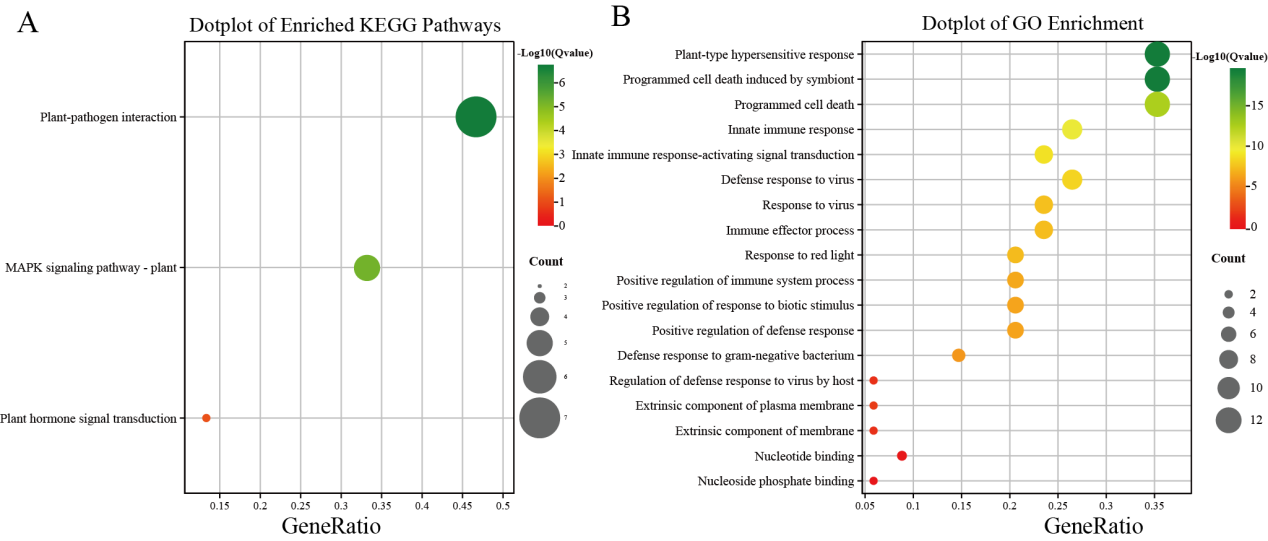


**Figure S7. The KEGG and GO enrichment analysis for** NBS **gene families for *L. ruthenicum.***  The enriched terms with adjusted *P* < 0.05 are presented. Color of the bubbles indicates statistical significance of the enriched terms; size of the bubbles indicates number of genes.


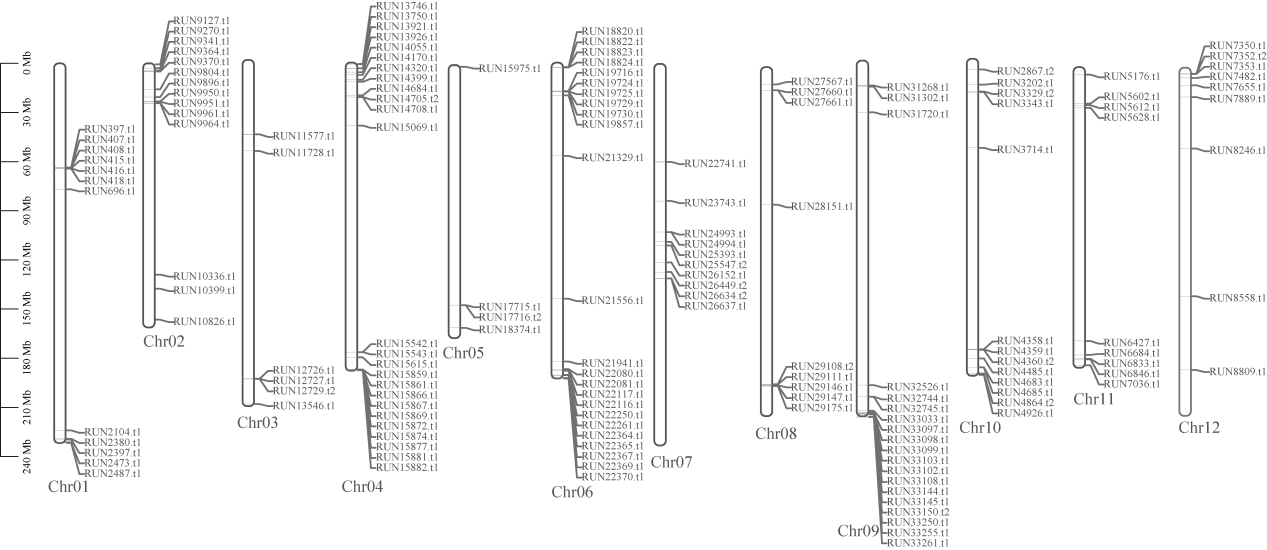


**Figure S8. Location of predicted 154** **NBS genes in the whole genome of *L. ruthenicum***
